# The London Medical Man

**Published:** 1881-10

**Authors:** Edward S. McKee

**Affiliations:** London


### Foreign Correspondence.

### Article XI.—The London Medical Man.

Messrs. Editors :—In these pages I will endeavor to describe to you, so far as lieth within me, the London Medical Man in all his stages, from all his aspects. I will touch especially on his education—preliminary and medical—his earlier years of practice, the divisions of practice, his finances, his way of doing business, his manner of living, discouragements, enjoyments and social position. I speak from five months residence in London, in daily contact with the London medical man and his work in the various hospitals, schools of medicine, and private practice, and his social world. Also, from having interviewed men in the various divisions of the practice, and the various stages of life; also those who look on and see from an impartial and unbiassed standpoint.

To have a good basis, we will allow that he is born at full term, the legal child of virtuous parents, a vertex of the 1st; that he cries lustily; that the cord is tied and cut *secundum artem;* that he is carefully washed, and, as soon as the doctor leaves, given butter and sugar, and, possibly, a "dhrop of spirits." All this he survives, as does he also, cake, buns, beer, pickles, and "just what we ourselves have;" conquering, also, the dessicating cord, the thrush, the ruthless vaccination, the perils of the first summer, teething, measles, whooping cough, scarlet fever, chicken-pox, mumps, knives and breeches. He lives on despite the school-board, the school-marm or master, the vitiated atmosphere of the school room. Rolls, rules, after hours and lessons affect him not. He is born to be a doctor, and not to die in infancy or childhood as do thirty per cent. of his fellows. He follows this schooling till the age of sixteen years. He becomes eligible to register and make his matriculation examination and enters the study of medicine. He either does this, or, as a minority do, goes on taking the degree of B.A., or B.Sc., at Oxford, Cambridge, London, or some other university.

Just here may be given the two great divisions and the four subdivisions of those who in the United States would come under the title of doctor. First are the physician and surgeon. Then the general practitioner of medicine, the consulting physician, the general practitioner of surgery, and the consulting surgeon. These four divisions are quite definite.

The general practitioner of medicine generally does not have a literary degree, but passes his matriculation examination at the Royal College of Surgeons, Royal College of Physicians, Apothecaries Hall, one of the Universities, or at the Hospital Medical School at which he enters, or one of the numerous other places. As an example of their nature I will quote the requirements of the matriculation of the Royal College of Surgeons: a certificate of having been registered by the General Medical Council; writing from dictation; English grammar; writing a short English composition; arithmetic, geography, English history, mathematics; algebra, through simple equations; Euclid, books I and II; translation from Cæsar de Bello Gallico. These are compulsory, besides which are a number of optional subjects. His studies are only recognized from the time of his registration. He may have studied any length of time before his name is placed on that public register but it is no good as to time. He then either goes to some recognized provincial hospital for eighteen months, or goes as the pupil of some legally qualified and registered practitioner for the same length of time. Said practitioner must be connected with a hospital, dispensary, or hold a poor-law appointment, so as to be enabled to give his pupil instruction. Not more than eighteen months of such study as the above is recognized by any of the licensing bodies. Such study is becoming more infrequent and unpopular, the student generally going immediately into his hospital medical school. It is strongly recommended that the pupil pass his preliminary examination before endeavoring to do anything at medicine, so as not to have too many irons in the fire. At least two and one half years of the four years required must be spent at a hospital medical school, including three winter and two summer sessions, the former six months the latter three months each. Thus the English *annus medicus* equals nine months. A hospital, to be recognized by the licensing bodies, must have 150 beds. All of the first and a greater part of the second year he spends with the bones, the anatomy, in the physiological laboratory and dissecting room, practical pharmacy, lectures on materia medica and botany, peeping occasionally into the wards and out-patients' rooms, and of course being a regular attendant on the "capital operations," and prepares for "the first college exam." This passed he enters regularly into the study of medicine. He clerks, or, as the English say "clarks," with the *a* as broad as you can make it. This he may do in either the wards, out-patients, or special departments. It consists of examining the patients, taking notes or histories, and generally assisting the physician in charge and endeavoring to answer all the questions put to him by said physician. This he must do for six months. He hears lectures higher in the scale, including medicine, surgery, midwifery and gynæcology, instruction and proficiency in vaccination, pathological anatomy, forensic medicine, and does post-mortem work. Having completed four years study under the above regulations, he is permitted to come up for his pass examination. A large per cent. fail the first time and are remanded for three or six months. There are nineteen licensing bodies in Great Britain, and he has his choice. None of the applicants for license are allowed to be examined by their own teachers. A majority do not get into practice for five years. The general practitioner after receiving his license generally settles down to business in some modest house in a modest street, and takes unto himself, probably a modest wife. He usually takes the license of the Royal College of Physicians, or the license of the Society of Apothecaries, with probably a surgical degree. During the first two or three years after this he takes the M.D. degree, from Oxford, Cambridge, London, Durham or some other of the universities.

As to the consulting physician the differential diagnosis is this:

He is a little more ambitious in the start and his ambition and patience grow and increase. He also usually commences with more money—the average student probably commencing with from $2,000 to $3,000. He takes the B.Sc. from the London University, or is a graduate in arts from some of the universities in the majority of cases. Usually spends five years before he takes his M.B., or M.R.C.P. Practice is a thing he does not bother about. He hangs around his hospital or other hospitals. If sufficiently influential, or expert enough in wire pulling, he gets a house physiciancy, may be a house surgeoncy, or he may go through both these positions, also that of midwifery, assistant or surgical registrar, or chloroformist. He looks out for assistancies in the special hospitals of various kinds. He endeavors to put in about three years in work of this kind, making in all probably eight years before his brass plate goes on his door. During these years his love for the study which first engaged his studious attention is paramount, and he takes on letters whenever possible. For instance, a young man who recently, in London, attained to a position of honor, writes after his name, A.M. M.B. (Cantab.), M.R.C.S., Eng., and lives. The love of the London medical man for the alphabet is wonderful.

The general practitioner of surgery and the consultant in surgery, differ from their brothers, the physicians, in their education only, as you can easily imagine. They pay more attention to surgery than to medicine, though they generally take a medical degree. The consultant, especially, is very jealous of his title of "Mister." If you wish to see his indignation rise just call him "Doctor." Consulting surgeons are Fellows of the Royal College of Surgeons. Throughout the lectures each man's presence or absence at each lecture is registered, and if he does not attend a good portion his papers are not signed, unless the student can, by a few judiciously placed pennies, bribe the man who keeps this register. Class "Exams" and "Grinds," are held with regularity. As to fees, the perpetual ticket entitling the holder to all the hospital practice and medical school work throughout, in one of the best hospitals in London, is 132 guineas ($670.56). This even does not entitle him to his degree, but he must pay, for the license of the Society of Apothecaries,

$30 ; for the license of the Royal College of Physicians, $76 ; for the membership of the Royal College of Surgeons, $107 ; and on failing to pass, a portion only of the fees are returned. The high fees are somewhat offset by large scholarships. These, varying in value from $250 to $700, are won annually by competitive examinations, the poor student's only chance. I might add, what does not appear in the prospectuses of the London hospital medical schools, but which forms, to the student at least, an important part of the curriculum, there are boat-racing, foot-ball, cricket, and lawn tennis. It might be well to add to the curriculu of the medical schools of the United States from the London schools, botany, forensic medicine, comparative anatomy, and mental diseases. It is not necessary to add boat-racing, cricket, etc. It would be well were the required time of medical study in the United States, instead of three years, four years as in London. It would be better were it also made a requirement that the students study with as much diligence throughout the four years as they do throughout the three. Occasionally, an Englishman goes abroad to study medicine, but not often, for there is a prevailing opinion here that what is not known in London is not worth traveling expenses. As an example of this opinion, a medical man of Edinburgh inaugurates a new system and secures its introduction and practice in Germany, France, America and elsewhere, yet is obliged to pack up his household plunder and come down to London, and become of London, before he can introduce his system in London. There is, however, at Oxford a scholarship, the taker of which must have spent eighteen months abroad.

Having the men now educated, we will start them into practice. The general practitioner of either medicine or surgery generally starts in pretty readily. He rents a house at $250, $1,200 or $2,000 a year, and commences. Rather oftener than otherwise, he is in partnership with one or two others, and the firm, if a good one, will make $10,000 to $30,000, less costs of medicine; one man alone, making the half or the third of this amount. His hours are rather irregular, being liable to be called at any time, bnt if alone he tries to keep some hours. If in a firm some one is generally at home. He does, as a rule, his own bookkeeping, and sends his bills out by mail quarterly or semi-annually. His fees range from 62½ cents to $2.50, with a preponderance towards the former.

The consultant's life is quite different. He thinks not of practice, but still hangs about his hospital medical school and special hospitals. He takes such positions as casualty physician, demonstrator of anatomy, practical surgery, tutors students, assists others, takes the places of members of the staff when absent, maybe gets a lectureship in the hospital medical school, next a situation as assistant physician or surgeon—his work in the out-patients' departments of the hospital. All this time his profession has brought him but the smallest stipend. After having been assistant physician to the hospital for probably fifteen years, some one above him dies, gets old, or angry, and he steps into his place. A consultant in London must hold a hospital position or his profession will amount to *nil.* During those years of trial—the first fifteen—he must keep up appearances, and seem, at least, to be making money. Many make something at other employments; for instance, writing articles for journals. Many have a private fortune to begin with, and their income keeps them. These terrible fifteen years he waits in his house, the best he can afford, and generally situated in the West End, and does not make $500 per annum. The surgeon generally works in sooner than the physician, for people would as willingly, perhaps more so, see a young man with good nerves coming at them, knife in hand, as an old, gray-headed man with glasses, and hands like an aspen leaf. The old surgeon often throws away the knife, and only gives opinions. A consultant always charges a guinea fee,—"a guinea or nothing." A few of the leaders, in order to "weed" a large practice, charge two guineas, and it has the desired effect. The consultant's fee is cash. He is called in, his opinion given, and the guinea taken. Such is the custom, and it is rarely broken. He keeps no books, collects no fees, writes no duns. The consulting physician is bound by his diploma not to go into partnership, nor into court for fees. Upon doing so he relinquishes his diploma, also his standing; hence, he never does it. Of this the laity, of course, are not generally aware. The consulting surgeon is really under no such restrictions; yet practically, from the general opinion of his fellows, he is as closely bound to the same rule as his brother the physican. A general practitioner of either medicine or surgery rarely grows into a consultant. In the occasions in which he does, he is expected to, as it were, wean himself from general practice by a season of total abstinence, as to practice, for two or three years. His office hours are, in the morning, until one P.M.; in the afternoon he goes out to see patients to whom he is called, usually going in a close carriage, with one horse and a coachman. The evening he spends in his study, or as seemeth to him best. In his money-making days, the consultant makes an average annual income of probably $6,000 to $15,000, with the leaders, who can be enumerated digitally, making $25,000 to $60,000 per annum. If called out of town he charges at the rate of $3.39 per mile—no more, no less. A few of the operating fees are these : calotomy, amputation hand or arm, $125 each ; amputation leg, $250 ; lithotomy, $250. The universal shingle is a brass door-plate, which is large proportionately to the want of knowledge on the part of the practitioner. Some men embark in dispensaries. Charitable dispensaries are generally considered honorable. Paying dispensaries are frequently a little crooked. Fees are low. For instance, the window will contain the following list of prices :

| | |
|---|---|
| Advice with medicine | $ .25 |
| Visits | .25 |
| Visits for one week daily | 1.00 |
| Midwifery, with attendance, one week | 1.25 |

Homœopaths thrive. There are not many, but what there are are well kept by the pampered class, the nobility, who can't bear to take nasty things. A homœopath must get regularly qualified through some of the qualifying institutions. One may make loud assertions that he intends to practice homœopathically, and present himself and get his qualification from a regular licensing body. There is a homœopathic school in London, but it cannot qualify.

With reference to the social standing of the medical men of London, I wish to express myself with great caution. Few have received from them more kindnesses, more courtesies and more favors than I, an utter stranger in a strange land. Few have for them a higher regard. Such being the fact, I hope and think it will keep me from doing them any injustice, or by mischievous words or hasty sentences conveying any impressions reflecting disparagingly against them.

In judging of their social status, one has carefully to bear in mind the peculiar forms of English society. Here men are classed after the most arbitrary fashion. One holds his social position not so much with reference to his merit and attainments as by certain accidents and adventitious relationships. Birth, kinship, wealth, title, station, even locality, chance surroundings do, as an American must think, decide most preposterously the social status of an individual. It is extremely difficult to fully understand this, unless one has resided in this country and closely studied its peculiar social conditions. The members of the medical profession form no exception to this idea—that all men are *not* born free and equal. A London medical friend recently stated to me that the physicians and surgeons in a certain portion of London, a large portion, are considered "pariahs, snobs, cads, kicked and cuffed by every one." A sad picture, drawn by no amateur hand in these few words. And yet there are men practicing in that area whose attainments, worth and refinement of character are such as to entitle them to the very highest social position in any country where society is not outlined by such whimsical distinctions as rule here.

This reminds me to call your attention to the fact that about two years ago a London correspondent for a Philadelphia medical journal got into rather hot water with the profession here by making a similar statement— indeed, saying in so many words that the medical man in London was a social pariah. This was at once hotly contested by some of the brethren here, but the writer maintained his ground vigorously, and I am inclined to think successfully. Undoubtedly the physician does not rank so high, socially, as he deserves, but this even requires a qualification. If the medical man be compared solely with those who hold their positions not merely by extreme wealth, titles or birth, but by education and merit, he would rank high. A few have been knighted, and are Sir Medicus, and their wife Lady Medicus, and walk out to dinner before their less honored fellows. In many cases, however, this knighthood is given with restrictions; for instance, that it cannot descend but by direct male line from father to son; no atavism allowed. Sir Medicus of course goes among the swells, who honor his presence, if not very heartily, at least by sufferance. He gets this title usually from having attended some member of the Royal family of this or some other kingdom, empire or principality in severe illness. Of course merit is presumable on his being chosen to attend in said illness. One London surgeon received his title for passing a catheter on the King of the Belgians. There are now six Sir Medici in London. The first medical man so honored was Sir Hans Sloane, in 1716. They are distinguished from the plebeians by wearing a little red band on their coat sleeves, or when out riding on their carriages. This is the symbol of all knights created since the union of the kingdoms. A created knight, too, of course is not on the same equality as one whose line runs from him who supplied James I with forty men armed and equipped, or with £1,000 to subdue the Ulster revolt, and thus getting his knighthood; not even with the batch created by Charles I to settle and mobilize Nova Scotia, which they took care not to do. Some claim that the medical man here in his various divisions ranks socially in the same scale with the various divisions of the legal profession. Many others say not; but that the rank is: clergy, law, medicine.

I am sure that the London medical profession in America would occupy a first rank, socially compared, in the eyes of his fellow men. The American medical man is much ahead of his London brother. The same is the case in Dublin and Edinburgh. In these two cities the medical man is often a social light compared to the London man. This can be explained somewhat by the fact of London being the largest and greatest city in the world, and of course the greatest men of all classes, to an extent, come here—while in Dublin and Edinburgh the medical men have not such strong competitors usually as in London. But then it is also true that London has the greatest, at least many of the greatest, lights in the medical profession.

In illustration of the position London medical men hold among their fellows, one fact. During the recent long pending of a bill for the abolition of vivisection, in Parliament, the entire medical profession of the United Kingdom, by petitions, by deputations, arguments, labored articles in the medical journals; by importuning of members, etc., etc., brought all the force it could command to prevent the passage of said bill. Their efforts resulted in a most humiliating failure. During the pending of a bill of like character in the Pennsylvania Legislature, two medical men from Philadelphia visited the Legislature, and by a statement before it completely snuffed out the whole affair.

Their compensation shows them to be held in low estimation by their fellow men. A gentleman of good practice in London, living at an expense of $5,000 per annum, will often furnish advice and medicine for 25 to 35 cents. Scores of highly competent medical men in London are struggling and will continue to struggle for years, scarcely able to keep the wolf from the door. It is simply impossible that a man in this condition should occupy a high social position. He could scarcely be considered other than a "pariah from society," which society makes a god of wealth, and bows with obsequious adoration before its idols of gold and of silver. Sometimes the long lane turns, if health and pluck endure. One of the most eminent men now in London scarcely earned his salt until he had passed his fortieth year. The case is different now. He is one of those who "weeds" his practice. A proper appreciation of the services of a doctor seems to be absent. The other day a "gentleman,"—the word is used in its most flexible sense—drove up to a surgeon's door with liveried driver and lackey, entered the office with a flourish, and presented an injured joint to be examined. This was done, and advice given, when this apparent gentleman threw five shillings ($1.25) on the table. "Pardon me, sir," said the surgeon, "you have made a mistake. My fee is one guinea." ($5.00). "Oh," he replied, "I never give a doctor more than that." "Well," said the surgeon, "then I shall be compelled not to receive anything." "Just as you please," was the reply, and scraping up the five shillings he coolly walked out. The surgeon had no alternative, and he got nothing.

Generally speaking, a professional man called to attend one of the nobility scarcely receives courtesies above those extended to a common tradesman, which courtesies here are not much. If the case is not urgent he must await the leisurely movements of his patron, and attend when asked. For this delay he receives no explanation or apology, nor, however kindly treated by this patron in his sick chamber, would the physician dare to recognize him first on the street. He might be the recipient of a most brutal snub from Sir Blue Blood. Occasionally one loses a little of his sympathy for the maltreated Medicus when he sees him obsequiously bowing and scraping and fawning to a titled patron, and at another time coldly, imperiously and unsympathizingly cutting up and snapping at, yelling at and browbeating some helpless, suffering, trembling out-door hospital patient. The contrast between these two pictures is as that between a California May and a Maine December. Charge this not all to the doctor himself, but let his "raising" have its full share.

All good practitioners have probably read in their boyhood the Sunday-school story in which an errand boy in a store was asked by his former fellow tradesmen, the boot-blacks, how he liked it now being bossed about. "Oh, very well," he replied, "I pass it off on the ashman."

To these peculiarities of English society aforementioned is this much due. "Our insular condition," of which the Englishman is so fond of speaking, meaning his water-belted isle, is carried out in the life of the medical man. He has no more than a half dozen friends, and these form his world. All above him he fears; all below him he despises. He is, in fact, as hard on others as others are on him, and his social ostracism is in many instances from this reason merited. It is the most difficult thing in the world to get a half dozen medical men on the witness stand to agree on one of the most salient of medical scientific facts. I may also cite an instance where a medical man went on the stand and swore that a brother of the same hospital staff did not know or detect consumption in its plainest form, a failure to do which would have been a disgrace to his newest "clark" or junior hospital walker. And this voluntarily.

And yet, let it be plainly understood, and remembered too, that there are no more patient, laborious, tender-hearted, thoroughly qualified men to be found anywhere than are many types of the London medical man. EDWARD S. McKEE, M.D.

LONDON, 27th July, 1881.

